# Skeletal plasticity in response to embryonic muscular activity underlies the development and evolution of the perching digit of birds

**DOI:** 10.1038/srep09840

**Published:** 2015-05-14

**Authors:** João Francisco Botelho, Daniel Smith-Paredes, Sergio Soto-Acuña, Jorge Mpodozis, Verónica Palma, Alexander O. Vargas

**Affiliations:** 1 Departamento de Biología, Facultad de Ciencias de la Universidad de Chile and FONDAP Center for Genome Regulation. Las Palmeras 3425, Santiago, Chile. 7800003

## Abstract

Most birds have an opposable digit 1 (hallux) allowing the foot to grasp, which evolved from the non-opposable hallux of early theropod dinosaurs. An important morphological difference with early theropods is the twisting of the long axis of its metatarsal. Here, we show how embryonic musculature and the onset of its activity are required for twisting of metatarsal 1 (Mt1) and retroversion of the hallux. Pharmacologically paralyzed embryos do not fully retrovert the hallux and have a straight Mt1 shaft, phenocopying the morphology of early tetanuran dinosaurs. Molecular markers of cartilage maturation and ossification show that differentiation of Mt1 is significantly delayed compared to Mt2-4. We hypothesize on how delayed maturation may have increased plasticity, facilitating muscular twisting. Our experimental results emphasize the importance of embryonic muscular activity in the evolutionary origin of a crucial adaptation.

A major morphological modification in the evolution of birds from dinosaurs was the acquisition of an opposable hallux (digit 1, D1), the perching digit^1^. The foot of early dinosaurs like *Herrerasaurus* had four functional digits, all of which articulated to the ankle (tarsus) (fig. 1a). In early tetanuran dinosaurs like *Allosaurus,* and in their modern bird descendants, the hallux has lost its proximal articulation to the ankle, and its metatarsal (Mt1) has a tear-like proximally tapering shape that establishes a rigid non-synovial articulation to metatarsal 2 (Mt2). However, unlike modern birds, in early tetanurans D1 was not opposable (fig. 1b,c). Thereafter, during the early evolution of birds, the hallux became opposable to the other digits, allowing the foot to grasp (fig. 1d). The opposable hallux came to be intimately associated with the way of life of many modern birds and its evolution is often discussed in the debate about the cursorial or arboreal origin of birds^2^.

Modern birds are unlike non-avian dinosaurs in that Mt1 of the hallux is twisted along its longitudinal axis. This torsion has been shown to be a key anatomical contributor to the opposability of the hallux^3^. The twisted Mt1 makes the lateral sides of the phalanx and claw face the medial side of the foot, inverting their orientation (fig. 1e). The straight, untwisted shape of Mt1 in dinosaurs and the earliest long-tailed birds like *Archaeopteryx* supports the conclusion that these taxa had not yet evolved an opposable hallux^6^.

Mesozoic birds closer than *Archaeopteryx* to modern birds include early short-tailed forms such as the Confuciusornithidae^7^ and the toothed Enantiornithes^8^. They present a Mt1 in which the proximal portion is visibly non-twisted, while the distal end is offset (“bent”) producing a unique “j-shaped” morphology. This morphology is arguably an evolutionary intermediate between the straight Mt1 of dinosaurs and the twisted Mt1 of modern birds, and conceivably allowed greater retroversion of Mt1 than *Archaeopteryx*.

Most discussion on the evolution of the perching digit has centered on the comparison of adult morphology across species, but little is known about development, which can provide crucial information. D1 in the avian embryo is initially not retroverted^9^, and therefore becomes opposable during ontogeny, but no embryological descriptions address the shape of Mt1, and no information is available on the mechanisms of retroversion.

Here, we studied the development of the hallux using Galliformes (Japanese quail and domestic chicken), one of the most basal clades of crown birds that actively use their hallux for perching. We determined when and how the digit changes its orientation in relation to embryonic musculature and cartilage differentiation. Using pharmacological muscular paralysis, we assessed the role of embryonic muscular activity on retroversion. The new embryological and experimental data shows how the development of the opposable hallux has important consequences for discussing its evolution.

## Results

### Re-orientation of the hallux during development

**Figure 2:**
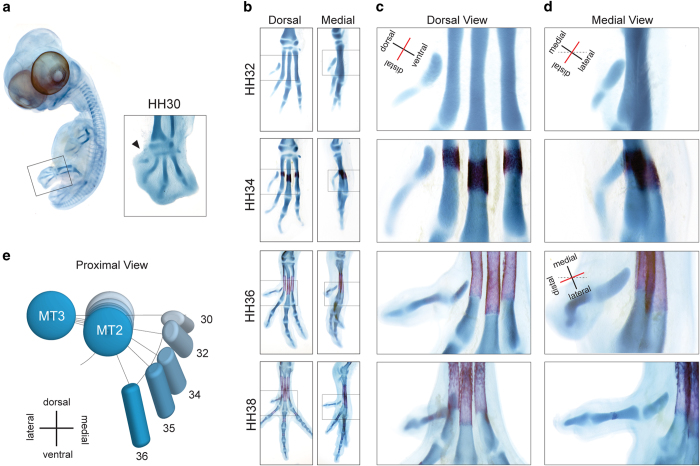
Re-orientation of the hallux. (**a**) Quail embryo stained with Alcian Blue, showing the small size and position of the hallux in the early stages of cartilage development (HH30); (**b**) Dorsal and medial views of Alcian Blue/Alizarin Red stained feet skeletons of quail embryos showing changes in the orientation of the hallux; (**c**) Details of hallux skeleton in dorsal view; (**d**) Details of hallux skeleton in medial view; (**e**) Diagram picturing the changing orientation of MT1 between HH32 and HH36 in relation to MT3 and MT2.

The cartilaginous precursor of Mt1 originates in the medial side of the foot, almost perpendicular to the limb’s main axis (HH30, fig. 2a, black arrowhead). A change in orientation of Mt1 begins at HH32, as the entire hallux becomes tilted, with its distal end slightly displaced towards ventral, and rotates such that its ventral side comes to face the lateral side of the foot, much like a human thumb (see HH32, fig. 2b-d). This orientation is similar to that of most non-avian tetanurans^1^. At stage HH36, D1 is re-oriented such that the entire hallux becomes perpendicular to the main limb axis, in an elevated, spur-like orientation (fig. 2b-d). Also at HH36, the proximal contact of Mt1 to Mt2 moves markedly towards the ventral face of Mt2 (fig. 2b-d). However, this does not cause retroversion: The ventral side of the hallux continues pointing towards the lateral side of the limb, with Mt1 abducted almost perpendicular to the tarsometatarsus. At HH38 an opposable hallux has been attained: It is no longer spur-like, presenting an opposable orientation (i.e., pointing towards ventral, fig. 2b-d) and its lateral side faces the medial side of the foot. Figure 2e summarizes the changes in the orientation of Mt1 with respect to Mt2 in successive stages of development.

### Maturation and ossification of Mt1 is delayed and begins at its proximal end

**Figure 3:**
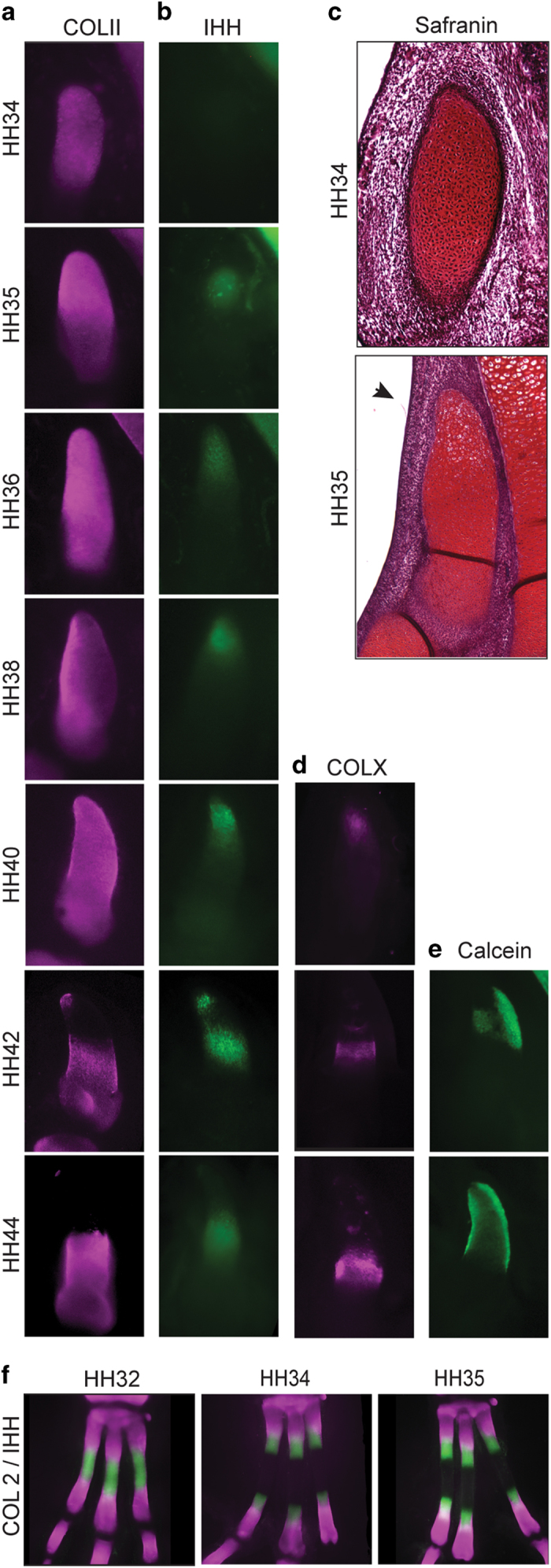
Arrested endochondral ossification of Mt1: (**a**) COLII expression during the development of Mt1 indicates the distribution of immature chondrocytes; (**b**) IHH expression during the development of Mt1 indicates the distribution of pre-hypertrophic chondrocytes; (**c**) Paraffin sections of Mt1 at HH34 and HH35 show the appearance of hypertrophic cells in the proximal side at HH35; (**d**) COLX expression during the development of Mt1 indicates the distribution of hypertrophic chondrocytes; (**e**) Calcein staining during the development of Mt1 showing the beginning of ossification; (**f**) Dorsal view of COLII (purple) and IHH (green) expression during the development of quail left metatarsi.

Embryonic cartilages are initially composed by immature chondrocytes, characterized by the production of collagen type IIA (COLII)^2^. Maturation of metatarsals 2,3 and 4 begins at mid-shaft as cells become flattened, and start to secrete Indian hedgehog protein (IHH)^4^. Those cells later exit the cell cycle, begin to grow (hypertrophy), stop producing COLII and synthetize collagen type X (COLX)^5^. Eventually, the hypertrophic cells die by apoptosis, leaving their extracellular matrix for ossification and remodeling. This dynamic of expansion from the center towards the ends generates two fronts of differentiation – the growth plates^6^. This maturation pattern was also the ancestral condition for Mt1, as observed in crocodylians (bird’s closest living relatives) and other amniotes^7^. The ossification of Mt2-4 begins around HH34 and their diaphyses are well ossified at HH37. In contrast with the other three avian metatarsals, Mt1 is a small element since the beginning of chondrogenesis (fig. 2a). The size difference increases at HH32, when the other metatarsi elongate conspicuously and Mt1 keeps its small elliptic shape. Differentiation of Mt1 is greatly delayed, and follows an unusual proximal-to-distal pattern. The presence of COLII as a marker of immature chondrocytes is detected in the entire element as late as HH38 (fig. 3a). In contrast, metatarsals 2, 3, and 4 exhibit large central regions with no COLII since HH34 (fig. 3f). IHH is clearly present in HH32 of other metatarsi but only becomes present in Mt1 at HH34, when its production starts in the proximal end (fig. 3b). Histological sections show that hypertrophic chondrocytes appear at the proximal end of the Mt1 at HH35 (fig. 3c). COLX synthesis begins in the proximal end of Mt1 at HH38 and advances towards distal in the next stages (fig. 3d). Calcification starts in the proximal end only at HH40 (SI, fig. S1), and the distal half of Mt1 is covered by collar bone at HH42 (fig. 3e)^0^. As a result of delayed differentiation from proximal to distal, Mt1 remains completely cartilaginous until exceptionally late (HH38) with a small diaphysis and just one growth zone in the distal end. Even after the beginning of ossification, the cartilaginous epiphysis continues to occupy about half its total length (HH42).

### The torsion of Mt1

**Figure 4:**
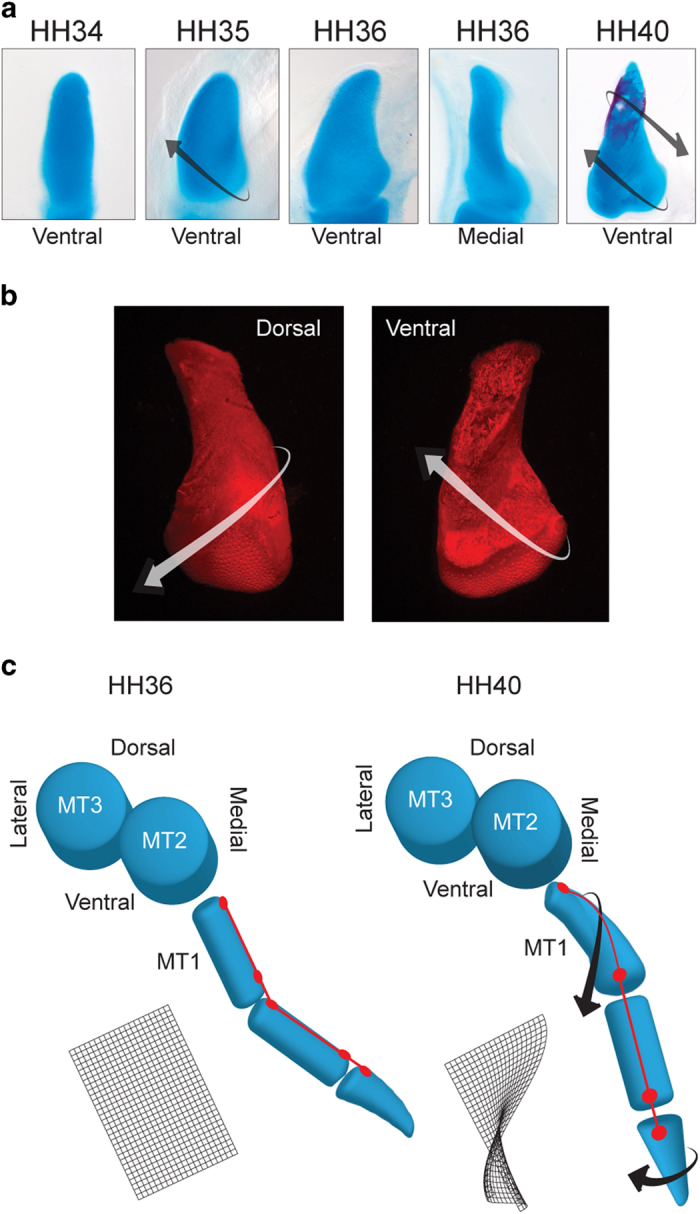
Torsion of MT1. (**a**) Alcian Blue/Alizarin Red stained Mt1 of chicken embryos showing the onset of its torsion. Arrows indicate the direction of torsion; (**b**) Alizarin stained Mt1 from a juvenile chicken in dorsal and ventral view; (**c**) Diagram picturing from proximal view the torsion orientation of Mt1.

In quail and chicken embryos at HH35, the long axis of Mt1 is straight. At HH36 it becomes slightly twisted at its proximal end, and at HH40 torsion is conspicuous along its entire axis (fig. 4a), similar to the completely ossified Mt1 of a juvenile chicken (fig. 4b). As described above, at HH36 the lateral side of the Mt1 is facing distal (figs. 2b-d and 4c, red line). The torsion of Mt1 is responsible for the change from this orientation to a completely opposable digit: as Mt1 twists, the lateral side of D1 changes from facing distal to facing medial (fig. 4c, red line). Throughout torsion (HH36-HH38) Mt1 is an immature, COLII-expressing cartilage (fig. 3a).

### Muscle development and activity in the hallux

**Figure 5:**
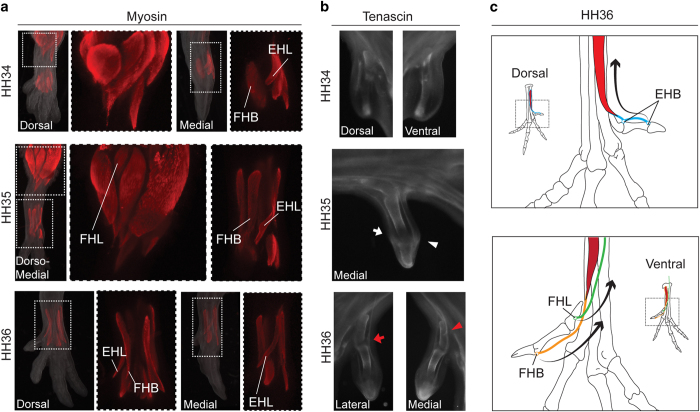
The early development of hind limb muscles and tendons: (**a**) Whole-mount immunofluorescence against myosin in quail embryos between HH34 and HH36 reveals the development of *musculus extensor hallucis longus* (EHL), *m. flexor hallucis longus* (FHL) and *m. flexor hallucis brevis* (FHB); (**b**) Whole-mount immunofluorescence against tenascin in quail embryos reveals the insertions of EHL (whitearrow), FHB (white arrowhead), and FHL (red arrowhead), and the secondary insertion of the EHL (red arrow); (**c**) Schematic representation of the foot musculoskeletal system at HH36 and the muscular forces proposed to provoke the twisting of Mt1.

Three muscles – two flexors and one extensor – control the avian hallux. The muscle *flexor hallucis longus* (FHL) is the only extrinsic muscle of the hallux – its belly is situated outside the foot, in the ventral shank; its tendon goes through the lateral hypotarsus and crosses obliquely to the ventral tarsometatarsus, and its principal insertion is in the ventral base of the ungual phalanx (fig. 5c). Quail embryos exhibit a well-individualized FHL at HH33, as visualized by whole-mount immunofluorescence against Myosin (fig. 5a). Its insertion is discernible by Tenascin expression at HH35 (fig. 5b, white arrowhead).

The other flexor of the hallux, the muscle *flexor hallucis brevis* (FHB), is located in the medio-ventral side of the foot; its tendon inserts in the ventral side of Mt1 (or at the base of the proximal phalanx, depending on the species)^1^. The muscle originates from a common mass of muscular fibre for all ventral intrinsic muscles. Its distal part is discernible since HH33 (fig. 5a); its insertion in the ventral Mt1 is visible at HH35 (fig. 5b, red arrowhead).

The *musculus extensor hallucis longus* (EHL) is the only extensor of the hallux. It arises from the dorso-medial border of Mt2 and its main insertion is in the base of the ungual phalanx. A secondary insertion in the base of the proximal phalanx is common. In quail embryos, it separates from the dorsal mass of intrinsic muscles at HH35 (fig. 5a), when its insertion is already visible (fig. 5b, white arrow); a secondary insertion is present at HH36 (fig. 5a, red arrow). Our data show that the musculoskeletal system is completely connected to the hallux at HH36, when torsion begins. The onset of digit movements occurs as soon as the muscular system is anatomically functional^2^. Digits are immobile until HH34, when the movements of the foot are restricted to the ankle joint. The first digit movements – synchronous extensions of all digits – appear at HH35, probably due to the action of the *musculus extensor digiti longus*. The flexion of the digits, including the hallux, starts at HH36 (see SI, Movie 1).

### The effect of muscular paralysis on hallux retroversion

**Figure 6:**
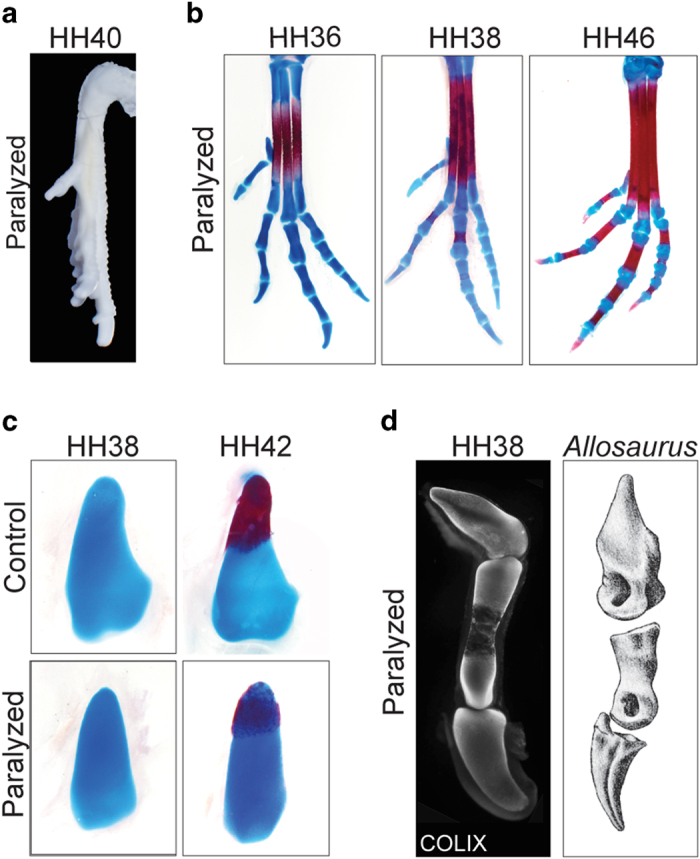
Paralyzed embryos fail to develop an opposable hallux: (**a**) Paralyzed chicken embryos at HH40 showing the maintenance of the medial orientation of the hallux; (**b**) Paralyzed chicken embryos exhibit the hallux articulated to the ventral Mt2 but not opposable; (**c**) Mt1 of paralyzed chicken embryos are straight, as opposed to control embryos; (**d**) Mt1 of paralyzed embryos at HH38 immunostained for COLIX resembles the non-twisted Mt1 of early tetanuran dinosaurs, as *Allosaurus.* (See SI, Movie 2).

The temporal dynamics of muscle development and activity suggests their involvement in the torsion of Mt1. Pharmacological and genetic impairing of embryonic muscular activity have been widely used to show its importance in shaping the vertebrate skeleton^8^. We tested the hypothesis that muscular activity is influencing Mt1 torsion by paralyzing chick embryos before the twisting of Mt1 (HH34). Paralyzed embryos show normal ventral displacement of Mt1 (fig. 6b), indicating that the change of the articulation site to Mt2 from medial to ventral is not influenced by muscular activity. Nevertheless, in paralyzed embryos the ventral side of the hallux faces the medial side of the foot and at HH40 its long axis lays parallel to the long axis of Mt2, similar to control embryos before the torsion of the hallux (fig. 6a,b). Consistent with this, Mt1 of paralyzed embryos fails to twist and has a straight shape (fig. 6c). Importantly, the morphology of Mt1 in paralyzed chick embryos is remarkably similar to that of early tetanuran dinosaurs like *Allosaurus*^9^ (fig. 6d), having a straight shaft and a ventral side facing the medial side of Mt2 (See SI, Movie 2).

## Discussion

It is well known that the skeleton of vertebrates is plastic in response to altered muscular activity^2^. Although even the adult skeleton can change as a result of processes such as bone remodeling, morphology is much more affected at juvenile and embryonic stages of active cartilage growth. Several experiments have demonstrated the great early plasticity of the vertebrate skeleton, and a few are known to induce changes that reverse or imitate specific evolutionary innovations. For instance, pharmacological embryonic paralysis leads to agenesis of the tibiofibular crest of birds (an innovation present since early theropod ancestors)^3^, while terrestrialization of juvenile *Polypterus* (Bichir) results in modifications of the pectoral girdle resembling those of early stem tetrapods^4^.

The development of the avian hallux provides a new clear-cut case of skeletal plasticity underlying a classically discussed evolutionary innovation. The musculoskeletal system controlling the hallux is in place and active at HH36, when torsion begins in the immature Mt1 cartilage. Pharmacological paralysis confirms that embryonic muscular activity is necessary for torsion and opposability of the hallux. Our embryological observations show that torsion of Mt1 is the chief cause of digit reversion: Displacement of the site of articulation of Mt1 to Mt2, from medial to ventral, does not affect the orientation of the hallux. Throughout the twisting process, Mt1 remains an immature, COLII-expressing cartilage, and torsion is over before chondrocytes even begin to express COLX, an indicator of mature cartilage. Differentiation and ossification of Mt1 is also notably delayed with regard to the other metatarsi. This suggests that Mt1 is especially plastic at the stages in which embryonic musculature produces twisting.

**Figure 7:**
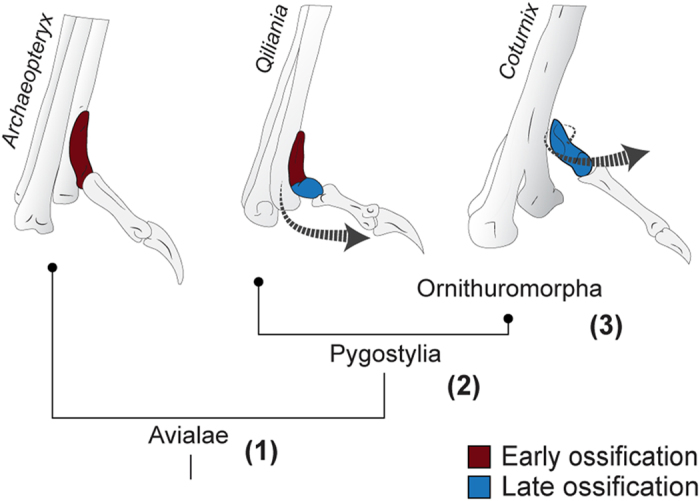
The evolutionary transition to a twisted Mt1. In Intermediate forms like the early short-tailed *Quiliania*, Mt1 has a non-twisted proximal end, but an offset (“bent”) distal end (arrow). Because Mt1 in modern birds matures from proximal to distal, we suggest that in (**1**) non-Pygostylian birds, Mt1 would have matured before the onset of muscular activity; (**2**) Maturation of Mt1 was delayed in pygostylia, allowing its distal bending by embryonic muscular activity; (**3**) Ornithuromorpha further delayed maturation of Mt1, allowing full torsion of its long axis by embryonic muscular activity.

Recent work has shown that embryonic muscular activity is required for retroversion of digit 4 in birds with zygodactyl feet^5^. Unlike “normal” feet with a non-retroverted digit 4, zygodactyl feet undergo asymmetric degeneration of the embryonic musculature of digit 4. This difference provides a straightforward explanation (unbalanced forces) for its retroversion. However, in the case of the hallux, the ancestral topology of musculature in tetanuran dinosaurs is inferred to have been similar to the topology found in modern birds^7^. If so, some additional factor must have been required for torsion of Mt1. Perhaps, delayed differentiation of Mt1 led to increased plasticity, allowing embryonic musculature to significantly alter its shape. Our data also shows that the differentiation of the hallux in modern birds follows a unique pattern where differentiation progresses from proximal to distal. In forms intermediate between *Archaeopteryx* and modern birds, the proximal end of MT1 is non-twisted, but the distal end is notably offset. The new context of developmental data suggests that muscle activity in these early Pygostylia could have encountered an already differentiated proximal end, but a still immature, more plastic distal epiphysis, leading to the characteristic J-like shape of their Mt1 (fig. 7). The hypothesis that successive delays in maturation allowed the progressive torsion of Mt1 is consistent with the fact that birds became increasingly paedomorphic since their dinosaur ancestors^8^ and that stem Maniraptora fossil embryos – including Enantiornithes – differ from extant birds in that they hatched with almost totally ossified metatarsals, metacarpals, and phalanges^3^. In an alternative scenario, embryonic motility may begin earlier or become more intense in modern birds than in their ancestors anticipating and/or amplifying the influence of muscles on Mt1 development. Interestingly, it has been shown that metabolism and rate of bone growth are correlated^4^, and that high metabolism in Aves depends on the increase of musculature to generate heat^5^, making it possible that both increased muscular activity and changes in skeletal development are physiological and evolutionarily related to each other through the increase in avian metabolism. The role of embryonic musculature also provides an explanation for secondary acquisition of a straight, non-twisted Mt1 in penguins and petrels: These are derived among modern birds in that the FHL and EHL muscles are absent (SI, fig. S2).

The hallux has been amply debated in terms of anatomical and adaptive evolution, but no data or experiments were available to include the mechanisms that generate discussed morphologies. Our developmental data provides an important missing piece of its evolutionary history, drawing attention to other potentially involved genetic and environmental factors. In particular, we show that changes in skeletal differentiation rates may secondarily enable influences of the muscular system, yielding a possible link with skeletal heterochrony and plasticity. The subject of skeletal plasticity brings up interesting theoretical questions, that are increasingly discussed in modern evolutionary theory. A common notion is that advantageous phenotypic plasticity (i.e., that allows the development of an adaptive trait) will tend to become canalized or genetically assimilated through natural selection^6^. However, the origin of a retroverted hallux is a case of acquisition of plasticity, rather than loss by genetic assimilation. Further, much like the formation of the fibular crest^3^, torsion of Mt1 in birds is phylogenetically ancient, but continues to develop based on mechanical forces, which have not been replaced/assimilated by some alternative genetic/molecular mechanism. Genetic assimilation of an adaptive trait may not be an inevitable outcome, especially so if development of the trait is based on systemic/epigenetic interactions that emerge reliably, in a recurrent and stable fashion^8^. In retrospective, we believe our data supports the need for a conceptual framework that encourages explicit discussion of developmental mechanisms and systemic interactions, further integrating this information with morphological evolution as observed in the fossil record^9^.

## Material and Methods

### (a) Experimental Animals and Nomenclature

Fertilized Broiler chicken and quail eggs were incubated at 37.5 °C and 70% humidity in an incubator with automatic rotating shelves. All animal procedures were in accordance with the Chilean legislation and were approved by Institutional Animal Care and Use Committees. We follow an anatomical terminology based on Baumel and Witmer^0^. The series of chicken and quail development are based on Hamburger and Hamilton (HH) stages^1^.

### (b) Skeletal Staining

Developmental series for quail and chicken were prepared for cartilage and bone staining. For cartilage staining embryos were fixed in 100% and stained in a 5:1 ethanol:acetic acid solution with 0.025% 8G Alcian Blue (Sigma-Aldrich) for two days at RT in an orbital shaker. For bone staining, embryos were post-fixed in 10% buffered formalin for 1 hour at 4 °C and stained with 0.04% Alizarin Red (Sigma-Aldrich) in 0.5% KOH for 1 hour at RT. Embryos were cleared in a sequence of 1:3, 1:1 and 3:1 glicerol:water. For fluorescent staining of bones, embryos were fixed in 4% of paraformaldehyde solution, washed and incubated for 2 hours in 10 μl/ml of Calcein (Sigma-Aldrich), and then cleared with Urea 4 M. Paraffin sections were cut at 7 μm thick and stained with Safranin and Hematoxylin employing standard histological protocols.

### (c) Whole Mount Immunofluorescence

Embryos were fixed in Dent’s Fix (4:1 methanol:DMSO) for 2 hours at RT, dehydrated in 100% methanol and left at −80 °C overnight. Before immunostaining, they were bleached in Dent’s Bleaching (4:1:1 methanol:DMSO:H_2_O_2_) for 24 h at RT. For anti-collagen immunostaining, embryos were dissected and digested with 2 mg/ml of hyaluronidase (Sigma) in PBS for 2 hour at 37 °C. Primary antibodies were diluted in 2% horse serum, 5% DMSO in PBST at the following concentrations: 1:20 anti-myosin 2 (MF-20, Developmental Studies Hybridoma Bank, DSHB); 1:10 anti-tenascin (M1-B4, DSHB); 1:40 anti-collagen type II (II-II6B3, DSHB); 1:20 anti-collagen type IX (2C2, DSHB); 1:20 anti-collagen type X (X-AC9, DSHB); and 1:200 anti-hedgehog (shh-160, Santa Cruz Biotechnology). Secondary antibodies anti-mouse (Alexa-488 and Alexa-Fluor 594, Jackson ImmunoResearch, PA) were diluted in 5% goat serum, 5% DMSO in PBST and incubated for 24 h at 4 °C in movement. Embryos were washed in PBST, cleared for five days in a solution of urea 4 M with 20% glycerol^2^ and then photographed in a fluorescent stereoscopic microscope (Nikon).

### (d) Embryo Paralysis

We treated seventy-two chicken embryos at HH34 in three independent rounds of experiments. Embryos were paralyzed by the application of 100 μl of a solution of 15 mg/ml of Decamethonium bromide diluted in PBS^4^. Control embryos received the same amount of the vehicle, PBS. The drug was delivered with a micropipette through a small hole in the shell above the air camera. Eggs were sealed with adhesive tape and re-incubated until the appropriate stages, verified by morphological criteria.

## Author Contributions

The authors have made the following declarations about their contributions: Conceived and designed the experiments: J.F.B., J.M., V.P. and A.O.V. Performed the experiments: J.F.B. and D.S.-P. Analyzed the data: J.F.B., D.S.-P., S.S.-A., J.M., V.P., and A.O.V. Wrote the paper: J.F.B. and A.O.V.

## Additional Information

**How to cite this article**: Francisco Botelho, J. *et al*. Skeletal plasticity in response to embryonic muscular activity underlies the development and evolution of the perching digit of birds. *Sci. Rep.* **5**, 9840; doi: 10.1038/srep09840 (2015).

## Supplementary Material

Supplementary Information

Supplementary Movie 1

Supplementary Movie 2

## Figures and Tables

**Figure 1:**
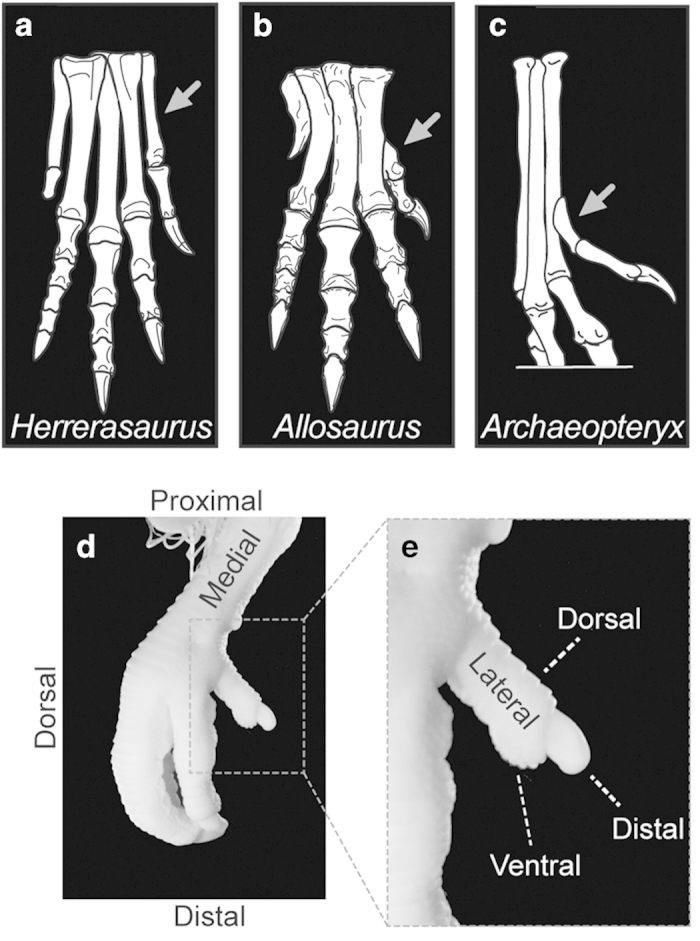
Major transformations of Mt1 in the evolution of avian opposable hallux. (**a**) Basal dinosaur exhibit the Mt1 (grey arrow) articulated to distal tarsals (*Herrerasaurus*); (**b**) Mt1 of basal tetanurans had lost its proximal epiphysis and articulated to Mt2 (*Allosaurus*); (**c**) Basal birds had the Mt1 articulated to the distal Mt2 (*Archaeopteryx*); (**d**) Most extant birds exhibit the hallux opposed to other toes, as observed in late chicken embryo (HH44); (**e**) Detail showing hallux inverted orientation.

## References

[b1] Sereno P. C. & Rao C. Early evolution of avian flight and perching: new evidence from the lower cretaceous of china. Science 255, 845–848, 10.1126/science.255.5046.845 (1992). 17756432

[b2] Padian K. & Chiappe L. The origin and early evolution of birds. Biological Reviews 73, 1–42, 10.1111/j.1469-185X.1997.tb00024.x (1998).

[b3] Middleton K. M. The morphological basis of hallucal orientation in extant birds. J. Morphol. 250, 51–60, 10.1002/jmor.1058 (2001). 11599015

[b4] Mayr G., Pohl B. & Peters D. S. A well-preserved *Archaeopteryx* specimen with theropod features. Science 310, 1483–1486, 10.1126/science.1120331 (2005). 16322455

[b5] Mayr G. & Peters D. S. The foot of *Archaeopteryx*: Response to Feduccia *et al.*(2007). The Auk. 124, 1450–1452 (2007).

[b6] Foth C., Tischlinger H. & Rauhut O. W. New specimen of *Archaeopteryx* provides insights into the evolution of pennaceous feathers. Nature 511, 79–82, 10.1038/nature13467 (2014). 24990749

[b7] Zhou Z. & Zhang F. Mesozoic birds of China—a synoptic review. Frontiers of Biology in China 2, 1–14 (2007).

[b8] Chiappe L. M, Walker C. A., Chiappe L. M. & Witmer L. M. in *Mesozoic birds: above the heads of dinosaurs* (eds Chiappe L. M. & Witmer L. M. ) 240–267 University of California Press 2002).

[b9] Heilmann G. The origin of birds. H. F. & G. Witherby1926).

[b10] Norell M. A. & Makovicky P. J. Important features of the dromaeosaur skeleton: information from a new specimen. American Museum Novitates 3215, 1–28 (1997).

[b11] Carrano M. T., Benson R. B. J. & Sampson S. D. The phylogeny of Tetanurae (Dinosauria: Theropoda). Journal of Systematic Palaeontology 10, 211–300, 10.1080/14772019.2011.630927 (2012).

[b12] St-Jacques B., Hammerschmidt M. & McMahon A. P. Indian hedgehog signaling regulates proliferation and differentiation of chondrocytes and is essential for bone formation. Genes & development 13, 2072–2086 (1999). 10.1101/gad.13.16.2072 PMC316949 10465785

[b13] Vortkamp A. *et al.* Regulation of rate of cartilage differentiation by Indian hedgehog and PTH-related protein. Science 273, 613–622, 10.1126/science.273.5275.613 (1996). 8662546

[b14] Kobayashi T. *et al.* PTHrP and Indian hedgehog control differentiation of growth plate chondrocytes at multiple steps. Development 129, 2977–2986 (2002). 12050144 10.1242/dev.129.12.2977

[b15] Minina E. *et al.* BMP and Ihh/PTHrP signaling interact to coordinate chondrocyte proliferation and differentiation. Development 128, 4523–4534 (2001). 11714677 10.1242/dev.128.22.4523

[b16] Kronenberg H. Developmental regulation of the growth plate. Nature 423, 332–336 (2003). 12748651 10.1038/nature01657

[b17] Reno P. L., McBurney D. L., Lovejoy C. O. & Horton W. E. Ossification of the mouse metatarsal: Differentiation and proliferation in the presence/absence of a defined growth plate. The Anatomical Record Part A: Discoveries in Molecular, Cellular, and Evolutionary Biology 288A, 104–118, 10.1002/ar.a.20268 (2006). 16342215

[b18] Mitgutsch C., Wimmer C., Sanchez-Villagra M. R., Hahnloser R. & Schneider R. A. Timing of ossification in duck, quail, and zebra finch: intraspecific variation, heterochronies, and life history evolution. Zoolog. Sci. 28, 491–500, 10.2108/zsj.28.491 (2011). 21728797 PMC3161728

[b19] Maxwell E. E. & Larsson H. C. E. Comparative ossification sequence and skeletal development of the postcranium of palaeognathous birds (Aves: Palaeognathae). Zoological Journal of the Linnean Society 157, 169–196, 10.1111/j.1096-3642.2009.00533.x (2009).

[b20] Maxwell E. E. Ossification sequence of the avian order anseriformes, with comparison to other precocial birds. J. Morphol. 269, 1095–1113, 10.1002/jmor.10644 (2008). 18496857

[b21] George J. C. & Berger A. J. Avian myology. Vol. 500 Academic Press 1966).

[b22] Gottlieb G. & Kuo Z.-Y. Development of behavior in the duck embryo. Journal of Comparative and Physiological Psychology 59, 183 (1965). 14288341 10.1037/h0021831

[b23] Kahn J. *et al.* Muscle contraction is necessary to maintain joint progenitor cell fate. Developmental cell 16, 734–743 (2009). 19460349 10.1016/j.devcel.2009.04.013

[b24] Blitz E. *et al.* Bone ridge patterning during musculoskeletal assembly is mediated through SCX regulation of Bmp4 at the tendon-skeleton junction. Dev Cell 17, 861–873, 10.1016/j.devcel.2009.10.010 (2009). 20059955 PMC3164485

[b25] Sharir A., Stern T., Rot C., Shahar R. & Zelzer E. Muscle force regulates bone shaping for optimal load-bearing capacity during embryogenesis. Development 138, 3247–3259, 10.1242/dev.063768 (2011). 21750035

[b26] Hall B. Genetic and epigenetic control of vertebrate embryonic development. Netherlands Journal of Zoology, *40* 1, 352–361 (1989).

[b27] Wu K., Streicher J., Lee M., Hall B. & Muller G. Role of motility in embryonic development I: Embryo movements and amnion contractions in the chick and the influence of illumination. Journal of Experimental Zoology 291, 186–194 (2001). 11479917 10.1002/jez.1068

[b28] Shwartz Y., Farkas Z., Stern T., Aszódi A. & Zelzer E. Muscle contraction controls skeletal morphogenesis through regulation of chondrocyte convergent extension. Developmental biology 370, 154–163 (2012). 22884393 10.1016/j.ydbio.2012.07.026

[b29] Madsen J. H. *Allosaurus fragilis*: a revised osteology. Utah Geological Survey Bulletin 109, 1–163 (1976).

[b30] Hall B. K. & Herring S. W. Paralysis and growth of the musculoskeletal system in the embryonic chick. Journal of Morphology 206, 45–56, 10.1002/jmor.1052060105 (1990). 2246789

[b31] Nowlan N. C., Sharpe J., Roddy K. A., Prendergast P. J. & Murphy P. Mechanobiology of embryonic skeletal development: insights from animal models. Birth Defects Research Part C: Embryo Today: Reviews 90, 203–213 (2010). 10.1002/bdrc.20184 PMC4794623 20860060

[b32] Kardon G. Development of the musculoskeletal system: meeting the neighbors. Development 138, 2855–2859, 10.1242/dev.067181 (2011). 21693508

[b33] Müller G. B. & Streicher J. Ontogeny of the syndesmosis tibiofibularis and the evolution of the bird hindlimb: a caenogenetic feature triggers phenotypic novelty. Anatomy and Embryology 179, 327–339, 10.1007/BF00305059 (1989). 2735527

[b34] Standen E. M., Du T. Y. & Larsson H. C. Developmental plasticity and the origin of tetrapods. Nature, 10.1038/nature13708 (2014). 25162530

[b35] Botelho J. F., Smith-Paredes D., Nuñez-Leon D., Soto-Acuña S. & Vargas A. O. The developmental origin of zygodactyl feet and its possible loss in the evolution of Passeriformes. Proceedings of the Royal Society B: Biological Sciences 281, 10.1098/rspb.2014.0765 (2014). PMC4083792 24966313

[b36] Hutchinson J. R. The evolution of hindlimb tendons and muscles on the line to crown-group birds. Comparative Biochemistry and Physiology-Part A: Molecular & Integrative Physiology 133, 1051–1086, 10.1016/S1095-6433(02)00158-7 (2002). 12485692

[b37] Dilkes D. W. Appendicular myology of the hadrosaurian dinosaur *Maiasaura peeblesorum* from the Late Cretaceous (Campanian) of Montana. Transactions - Royal Society of Edinburgh 90, 87–125 (1999).

[b38] Bhullar B. A. *et al.* Birds have paedomorphic dinosaur skulls. Nature 487, 223–226, 10.1038/nature11146 (2012). 22722850

[b39] Kundrát M., Cruickshank A. R. I., Manning T. W. & Nudds J. Embryos of therizinosauroid theropods from the Upper Cretaceous of China: diagnosis and analysis of ossification patterns. Acta Zoologica 89, 231–251 (2008).

[b40] Norell M. A., Clark J. M. & Chiappe L. M. An embryonic oviraptorid (Dinosauria: Theropoda) from the Upper Cretaceous of Mongolia. American Museum Novitates 3315, 1–20 (2001).

[b41] Varricchio D. J., Horner J. R. & Jackson F. D. Embryos and eggs for the Cretaceous theropod dinosaur *Troodon formosus*. Journal of Vertebrate Paleontology 22, 564–576 (2002).

[b42] Elzanowski A. Embryonic bird skeletons from the Late Cretaceous of Mongolia. Palaeontologia Polonica 42, 147–176 (1981).

[b43] Cheng Y. -n., Qiang J. I., Wu X.-c. & Shan, H.-y. Oviraptorosaurian Eggs (Dinosauria) with Embryonic Skeletons Discovered for the First Time in China. Acta Geologica Sinica - English Edition 82, 1089–1094, 10.1111/j.1755-6724.2008.tb00708.x (2008).

[b44] Grady J. M., Enquist B. J., Dettweiler-Robinson E., Wright N. A. & Smith F. A. Evidence for mesothermy in dinosaurs. Science 344, 1268–1272 (2014). 24926017 10.1126/science.1253143

[b45] Newman S. A., Mezentseva N. V. & Badyaev A. V. Gene loss, thermogenesis, and the origin of birds. Annals of the New York Academy of Sciences 1289, 36–47 (2013). 23550607 10.1111/nyas.12090

[b46] Waddington C. H. Genetic Assimilation of an Acquired Character. Evolution 7, 118–126 (1953).

[b47] Maturana H. & Mpodozis J. The origin of species by means of natural drift. Rev. Chil. Hist. Nat. 73, 261–300 (2000).

[b48] Griffiths P. E. in *Evolution and learning: The Baldwin effect reconsidered* (eds Weber B. H. & Depew D. J. ) 193–215 MIT Press2003).

[b49] Raff R. A. Written in stone: fossils, genes and evo-devo. Nat. Rev. Genet. 8, 911–920, 10.1038/nrg2225 (2007). 18007648

[b50] Baumel J. J., King A. S., Breazile J. E., Evans H. E. & Vanden Berge J. C. Handbook of avian anatomy: nomina anatomica avium. Publications of the Nuttall Ornithological Club 1993).

[b51] Hamburger V. & Hamilton H. L. A series of normal stages in the development of the chick embryo. J. Morphol. 88, 49–92, 10.1002/jmor.1050880104 (1951). 24539719

[b52] Hama H. *et al.* Scale: a chemical approach for fluorescence imaging and reconstruction of transparent mouse brain. Nat. Neurosci. 14, 1481–1488 (2011). 21878933 10.1038/nn.2928

[b53] Hall B. K. A simple, single-injection method for inducing long-term paralysis in embryonic chicks, and preliminary observations on growth of the tibia. Anat. Rec. 181, 767–777, 10.1002/ar.1091810408 (1975). 1119705

[b54] Pitsillides A. Early effects of embryonic movement: “a shot out of the dark”. Journal of Anatomy 208, 417 (2006). 16637868 10.1111/j.1469-7580.2006.00556.x PMC2100206

