# Supplementary material for: Skeletal plasticity in response to embryonic muscular activity underlies the development and evolution of the perching digit of birds
Source: Sci Rep. 2015 May 14;5:9840. doi: 10.1038/srep09840 (PMC4431314; doi:10.1038/srep09840)
Supplement: Supplementary Information [file srep09840-s1.pdf]

Supplementary Information

Skeletal plasticity in response to embryonic muscular activity underlies the development and evolution of the perching digit of birds

João Francisco Botelho[1]

Daniel Smith-Paredes

Sergio Soto-Acuña

Jorge Mpodozis

Verónica Palma

Alexander O. Vargas[1]

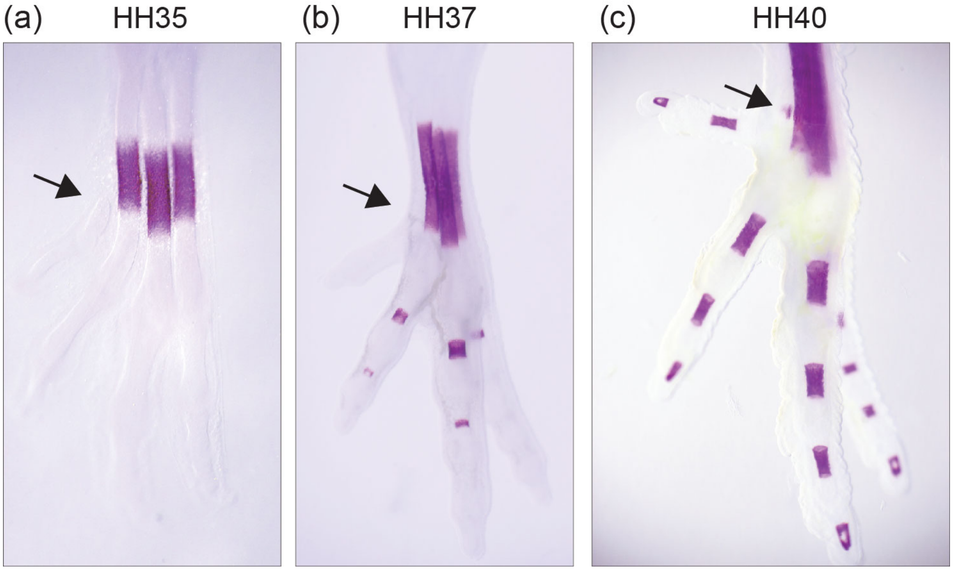

Figure S1: MT1 ossifies late in relation to other foot bones: Red alizarin staining of quail embryonic foot shows that the ossification of Mt1 (black arrow) is delayed in relation to other metatarsals; (A) HH35, (B) HH37, and (C) HH39.

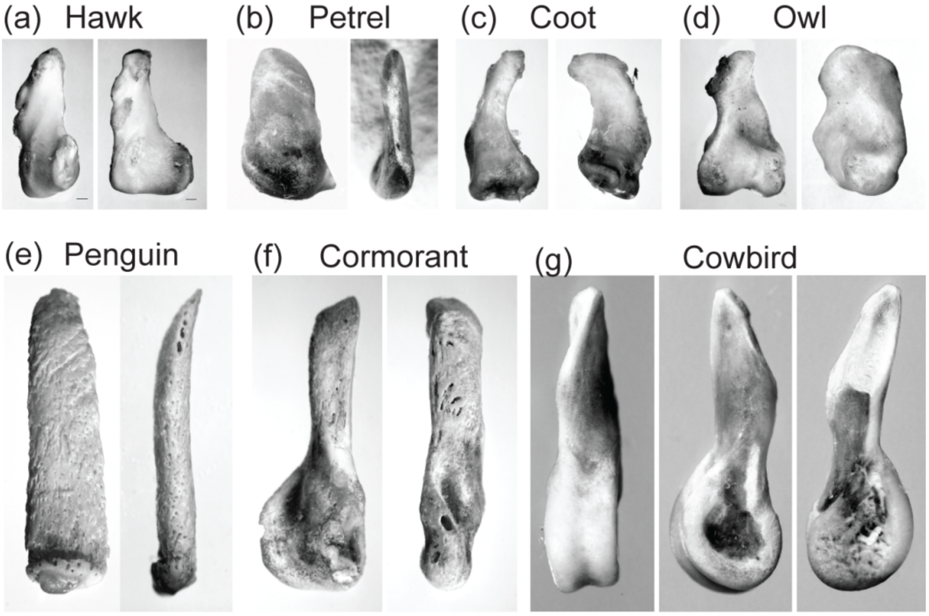

Figure S2: Variation of Mt1 shape in extant birds: The morphology, in different views, of the Mt1 of (a) Red-backed hawk (*Geranoaetus polyosoma*); (b) a petrel (*Puffinus sp.*); (c) Red-fronted coot (*Fulica rufifrons*); (d) Magellanic horned owl (*Bubo magellanicus*); (e) Humboldt penguin (*Spheniscus humboldti*); (f) Neotropic cormorant (*Nannopterum brasilianus*); (g) Shiny cowbird (*Molothrus bonariensis*). Modern petrels and penguins have secondarily re-evolved a straight Mt1. In these taxa, the FHL and EHL muscles are absent [1-3], which is consistent with the role of muscular activity in the development of the shape of Mt1.

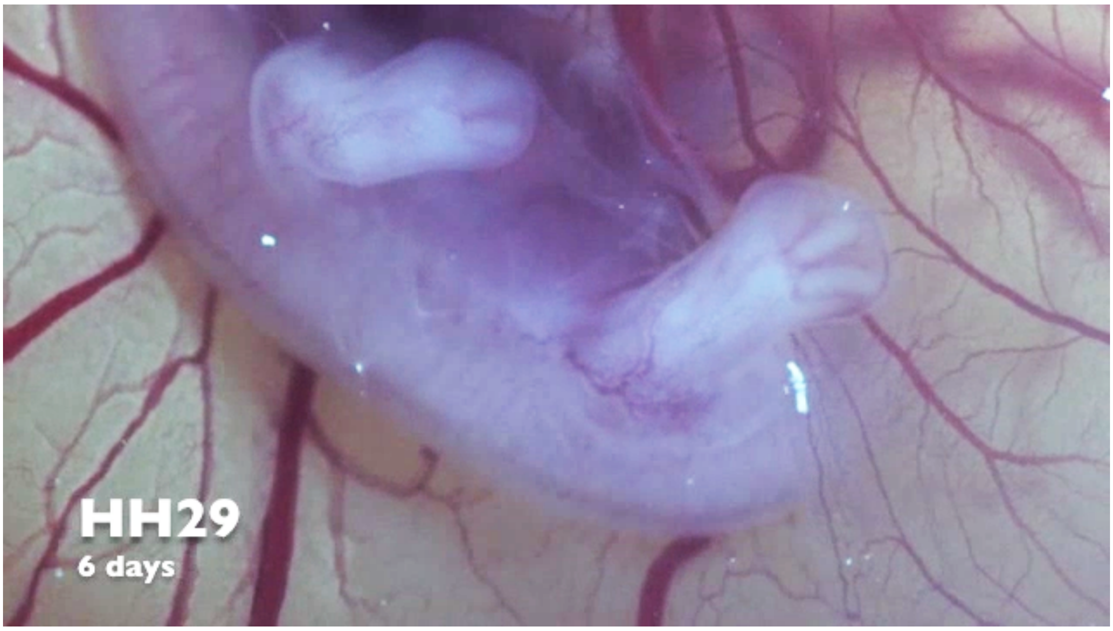

Movie S1: The early development of hind limb movements. Hind limb movements of chicken embryos inside the egg between 6 and 10 days of incubation. Hallux flexion starts at day 10 (HH36).

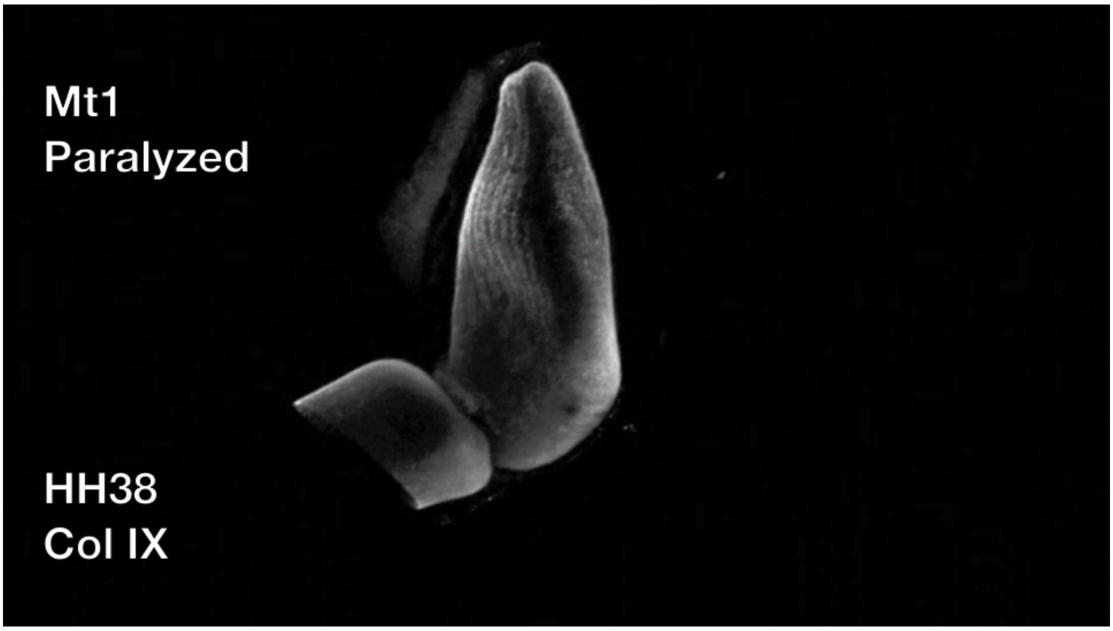

Movie S2: Confocal reconstruction of Mt1: COL IX immunostained control and paralyzed Mt1 at HH38 show that paralyzed embryos have a straight shaft, morphologically similar to early theropods.

References

1 George, J. C. & Berger, A. J. *Avian myology*. Vol. 500 (Academic Press, 1966).

2 Hudson, G. E. Studies on the Muscles of the Pelvic Appendage in Birds. *Am Midl Nat* **18**, 1-108 (1937).

3 Raikow, R. J. in *Form and function in birds* Vol. 3 (eds A. S. King & J. McLelland) 57-147 (Academic Press, 1985).
